# Mental health treatment among transgender and gender diverse people following gender affirming hormone therapy: evidence from whole-of-population Australian administrative data

**DOI:** 10.1016/j.eclinm.2026.103765

**Published:** 2026-02-02

**Authors:** Karinna Saxby, Tom Buchmueller, Christopher S. Carpenter, Clue Coman, Brendan J. Nolan

**Affiliations:** a Melbourne Institute: Applied Economic & Social Research, Faculty of Business and Economics, Room 5.55, Level 5, FBE Building, 111 Barry Street, Carlton, The University of Melbourne, Victoria, 3010, Australia; b Centre for Health Economics, Monash Business School, Monash University, Australia; c Ross School of Business, University of Michigan Ann Arbor, United States; d Department of Economics and Vanderbilt LGBTQ+ Policy Lab, Vanderbilt University, United States; e Centre for Social Research in Health, University of New South Wales, New South Wales, Australia; f Trans Health Research Group, Medicine (Austin Health), University of Melbourne, Victoria, Australia

**Keywords:** Transgender, Mental health, Gender affirming hormone therapy, Mental healthcare, Antidepressants

## Abstract

**Background:**

Gender affirming hormone therapy (GAHT) is associated with improved mental health outcomes among transgender and gender diverse (‘trans’) individuals, yet limited evidence examines how mental healthcare utilisation changes following GAHT initiation.

**Methods:**

Using Australian administrative data (2012–2024), we identified trans people initiating oestradiol-based GAHT (e-GAHT) or testosterone-based GAHT (t-GAHT). We applied a dynamic difference-in-differences model to estimate within-individual changes in mental health services and prescriptions (antidepressants, anxiolytics), using future GAHT recipients as controls. Effects were estimated separately by regimen and relative to individuals’ utilisation three years before GAHT initiation, up to five years post-initiation, and stratified by age (15–24, ≥25 years) and baseline mental healthcare engagement (above/below mean mental health prescription use).

**Findings:**

20,358 individuals initiated e-GAHT and 11,883 initiated t-GAHT (mean follow-up 4.1 and 4.9 years, respectively). Prior to initiation, e-GAHT recipients had lower engagement with mental healthcare than t-GAHT recipients. For both regimens, mental healthcare use rose at initiation but declined sharply thereafter, with larger initial increases for e-GAHT recipients. Five years post-initiation, e-GAHT and t-GAHT recipients used 0.29 (95% CI −0.03; 0.60) and 2.59 (95% CI 1.87; 3.31) fewer mental health services, respectively. Mental health prescription use among e-GAHT recipients initially rose and then fell to 0.53 (95% CI 0.20; 0.86) at five years, while t-GAHT recipients used 1.02 (95% CI 0.31; 1.72) fewer prescriptions after five years. Reductions in mental healthcare were more pronounced for individuals with higher baseline mental healthcare engagement as well as older e-GAHT recipients.

**Interpretation:**

Consistent with assessment and support requirements, mental healthcare use increases around the time of GAHT initiation—particularly among individuals with limited prior mental healthcare engagement and younger e-GAHT recipients—then declines substantially over time. Altogether GAHT may help address unmet mental health needs and contribute to longer-term reductions in mental healthcare use and associated costs among trans people.

**Funding:**

This work is supported by the University of Melbourne McKenzie Fellowship (2025MCK182), a Viertel Charitable Foundation Clinical Investigator Award, and a National Health and Medical Research Council Investigator Grant (2034450).

**Research in context**

**Evidence before this study**

We searched PubMed and Scopus via the University of New South Wales Library from database inception to July 29, 2025, using a structured search strategy restricted to terms appearing in the title or abstract fields. The search terms were: ((“gender identity”) OR trans OR transgender OR “non-binary” OR nonbinary OR “gender nonconforming” OR “gender diverse” OR “gender-diverse” OR genderqueer OR “gender queer”) AND (“hormone therapy” OR “hormone treatment” OR “gender-affirming hormone therapy” OR “gender-affirming care” OR “gender-affirming treatment” OR GAHT) AND (“mental health care” OR “mental healthcare” OR “mental health treatment” OR “mental health services” OR “psychiatric care” OR psychotherapy OR “psychological treatment” OR antidepressant∗ OR anxiolytic∗ OR “SSRI” OR “SSRIs” OR “selective serotonin reuptake inhibitor” OR psychotropic∗ OR mental health diagnos∗). This search returned 84 studies from PubMed and 99 studies from Scopus. After screening, we identified three studies directly relevant to our research question; i.e., mental healthcare use among trans and gender diverse (‘trans’) people receiving gender affirming hormone therapy (GAHT). While our scoping review identified a wider body of research assessing GAHT initiation and self-reported mental health outcomes among trans populations, there is limited population-level evidence exploring within-individual variation in the levels of mental healthcare utilisation (including mental health services use in addition to scripts), relative to GAHT initiation.

**Added value of this study**

Drawing on administrative data on the largest population-based study of GAHT recipients globally, we investigate changes in mental treatment following GAHT initiation for 20,358 e-GAHT recipients and 11,883 t-GAHT recipients in Australia. We apply a dynamic difference-in-differences approach, using future GAHT recipients as controls, enabling us to estimate within-individual changes in mental health service and prescription medicine use for up to five years after GAHT initiation. This design accounts for time-varying confounding and unobserved time-invariant confounders between individuals, significantly building upon previous studies. Further, our data provide extensive pre- and post- GAHT observation periods, allowing us to capture changes in the levels of mental health service and prescription medicine use relative to individuals’ baseline levels of mental healthcare engagement. Our findings suggest that GAHT may play an important role in reducing mental health treatment needs over time.

**Implications of all the available evidence**

Using information from the largest population-based study of GAHT recipients globally, we show that increased time since GAHT initiation is associated with reductions in mental healthcare use. Enhancing public access to gender affirming care for trans Australians should be considered to improve mental health and reduce associated healthcare costs. Restrictions on GAHT access may have long term deleterious mental health effects for trans people.

## Introduction

Emerging research has shown that compared to cisgender males and females, trans and gender diverse (‘trans’) populations are at much higher risk of mental health disorders, self-harm behaviour, and suicide.^1^^,^^2^ This elevated risk is widely attributed to social transphobia and other stigma-related stressors.^3^ Mental health inequalities can also be influenced by gender dysphoria, which refers to the distress that arises when an individual's gender identity does not align with their sex recorded at birth.^4^

Gender affirming medical care can be used to facilitate development of physical characteristics to align with an individual's gender identity. For some trans adolescents, this can involve puberty suppression, and then typically involves gender affirming hormone therapy (GAHT) and, for some individuals, gender affirming surgical interventions. Access to, public subsidisation of, and even the legal status of gender affirming care varies significantly across countries and jurisdictions within countries.^5^ In Australia, parenteral or transdermal testosterone is most prescribed for individuals desiring masculinisation, whereas oral or transdermal oestradiol is typically administered with an anti-androgen (spironolactone or cyproterone acetate) for individuals desiring feminisation.^6^

Existing studies, including prospective cohort designs, a recent randomised controlled trial, and quasi-experimental studies, suggest that GAHT is associated with better quality of life, decreased depression, and decreased anxiety, with better outcomes among individuals initiating at younger ages and among those receiving testosterone-based GAHT (t-GAHT) rather than oestradiol-based GAHT (e-GAHT).^7^, ^8^, ^9^, ^10^, ^11^ Of note, a randomised controlled trial showed that t-GAHT led to reduced gender dysphoria, depression, and suicidality^8^ and a quasi-experimental study, using future initiators as a comparison group, found reductions in suicide attempts following GAHT access among trans adolescents.^10^ However, due to data limitations, few studies have empirically investigated the longer-term effects of GAHT on mental healthcare utilisation. To our knowledge, only three studies have explored this relationship.

A cross sectional analysis of individuals diagnosed with gender incongruence in Sweden (n = 1885) found no statistically significant association between time since GAHT initiation and mental health treatment—including mood and anxiety disorder-related visits, prescriptions for antidepressants or anxiolytics, and suicide-related hospitalisations.^12^ Another study of trans adolescents in U.S. military connected families who initiated gender-affirming hormones (puberty blockers or GAHT) (n = 963) found no significant change in mental healthcare visits over a relatively short follow-up (median 1.5 years), but did observe an increase in psychotropic medication use.^13^ A Danish study of individuals with gender incongruence identified through national hospital registers found that, relative to a matched cisgender population, GAHT was associated with increased odds of receiving a prescription for psychotropic medication at one, five, and eight years following initiation, relative to two years before.^14^ This study also stratified results by regimen, finding similar patterns among e-GAHT recipients (n = 1043) and t-GAHT recipients (n = 1046).

Altogether, there remains limited population-level evidence investigating within-person changes in levels of mental healthcare use relative to e-GAHT or t-GAHT initiation. Moreover, to our knowledge, no prior studies have examined heterogenous effects by age at initiation or baseline mental healthcare engagement.

In this paper, we contribute to the evidence base by leveraging whole-of-population administrative data which captures all trans Australians who accessed government-subsidised GAHT between 2012 and 2024. This provides a unique opportunity to investigate the dynamic effects of GAHT initiation over an extended period. Consistent with Australian treatment guidelines recommending concurrent access to mental health support at the time of GAHT initiation,^6^ we hypothesise that GAHT will be associated with an initial increase in mental healthcare utilisation. However, given that initiation of GAHT has been associated with reduced gender dysphoria and improved mental health,^7^, ^8^, ^9^ we anticipate that, in the longer term, GAHT will be associated with a reduction in the use of both mental health service and mental health prescriptions.

## Methods

### Data

The data for this analysis comes from the Person-Level Integrated Data Asset (PLIDA), an individual-level linked dataset that combines information from population Census and various administrative data sources including healthcare records, social security records, income, and death records.^15^ Healthcare records capture publicly subsidised out-of-hospital healthcare services and prescription medications for all Australian citizens and permanent residents (provided under Australia's universal health insurance scheme, Medicare). For this study, we sourced data from April 2012 to December 2024 (the most recent and complete data available at the time of writing). Because this study used de-identified administrative data accessed through the Australian Bureau of Statistics' DataLab, individual informed consent was not required under the National Statement on Ethical Conduct in Human Research.

### Classification of trans population initiating GAHT

The prescription medication records contain information on an individual's ‘gender marker.’ Historically, this marker, which is recorded as male or female (and therefore does not capture non-binary or other diverse gender identities) was categorised based on sex at birth. Although individuals may update their gender marker in Medicare (since 2013), the markers are still binary (i.e., there are only ‘male’ or ‘female' options).

Following the previous literature,^16^^,^^17^ we identified trans people accessing GAHT using information on gender markers and hormone therapy prescriptions. Namely, trans people accessing t-GAHT were classified as those who ever used testosterone and had a current or previous female gender marker and trans people accessing e-GAHT were classified as those who ever used oestradiol and had a current or previous male gender marker.

In line with previous research, individuals taking both oestradiol and testosterone were excluded to avoid ambiguity in e-GAHT vs t-GAHT assignment.^18^ To increase confidence that we were observing individuals’ initiation of GAHT, we also excluded individuals who were already receiving GAHT in 2012. In addition, as mental healthcare treatment, subsidisation, and use guidelines differ for children in Australia,^19^ we excluded all individuals that initiated GAHT before age 15.

### Outcome measures

Mental health services included all Medicare-subsidised out-of-hospital services including mental health services provided by general practitioners, psychiatrists, psychologists, and other allied health professionals.^20^ Mental health prescriptions were identified based on the Anatomical Therapeutic Chemical (ATC) classes for antidepressants (‘N06A’) and anxiolytics (‘N05B’). For each individual, we aggregated outcomes at the annual level.

### Statistical analyses

We first reported characteristics of the sample for individuals initiating e-GAHT and t-GAHT between 2013 and 2024. To describe the patterns of mental healthcare use for trans Australians relative to initiating GAHT, we then estimated an event study difference-in-difference model^21^ that exploits variation in the time in which individuals initiated GAHT. In this model, each person's exposure changes when they reach their initiation date; each individual contributes unexposed time until their initiation date, and exposed time afterward.

Namely, we estimated means of outcomes (number of mental health services and mental health prescriptions per person per year) among individuals that had initiated GAHT compared to those who had not yet initiated GAHT, after confounder adjustment. We controlled for individual fixed effects to account for all observed and unobserved time-invariant confounders. These include demographic and baseline characteristics that do not change over time, such as country of birth, sex assigned at birth, age at hormone therapy initiation, and other stable factors like underlying health predispositions or personality traits.

To account for secular changes that may have impacted mental health service use and prescribing over time, we included a full set of calendar year fixed effects (i.e., indicator variables for each observation year). This approach does not impose a functional form on time, instead it flexibly captures both linear and nonlinear time trends common to all individuals (e.g., secular changes that could impact mental health or access to healthcare over time, such as national policy changes or broader economic conditions). The event study was estimated separately by regimen type (i.e., t-GAHT or e-GAHT) for five years before and after GAHT initiation, with all event-time coefficients referenced to three years before GAHT initiation (i.e., event time = −3). Thus, individuals become ‘exposed’ from two years before GAHT initiation. This approach was chosen as, in alignment with clinical guidelines,^6^ we may observe that individuals consult a mental health professional before initiation of treatment. This reference period also follows similar event-study analyses in this space which acknowledges that gender affirmation is a multi-year process that may begin several years prior to initiating GAHT.^22^

We then conducted heterogeneity analyses. First, we stratified the results by age of GAHT initiation (15–24 years and 25 years and above). These age bands were selected to align with the World Health Organization definition of ‘youth’ as people aged 15–24 years.^23^ This broad age band selection also ensures sufficient longitudinal observation time and statistical power within each stratum. For example, as our sample comprises individuals initiating at 15 years and above, individuals initiating GAHT at younger ages (e.g., 15–17 years) have limited pre-treatment periods available in the dataset. We then stratified results by individuals' baseline engagement with mental healthcare. Specifically, we defined baseline engagement based on whether individuals' annual use of mental health prescriptions was below or above the average for their regimen in the years before the reference period.

We reported point estimates and 95% CIs from these models. The empirical model and inference approach is provided in the Supplementary Material SM1. All analyses were conducted using STATA version 18. No primary data collection was undertaken. Data from PLIDA was analysed as per the data-sharing agreement with the Australian Bureau of Statistics. By construction, there were no missing data for exposures, covariates, or outcomes. Individuals were followed continuously in linked national datasets and were censored only at death.

This study is reported according to STROBE guidelines.

### Role of the funding source

Funding bodies had no role in study design, data collection, data analysis, data interpretation, or writing of the report.

## Results

### Descriptive statistics

The descriptive statistics for the study sample are presented in Table 1. Between 2013 and 2024, 11,883 individuals initiated t-GAHT and 20,358 initiated e-GAHT. On average, t-GAHT recipients were younger at initiation than e-GAHT recipients (25 vs 34 years) and were more likely to initiate in more recent years. For example, 35% of the t-GAHT sample initiated in 2023 or 2024, compared to 29% of the e-GAHT sample. In contrast, a greater share of e-GAHT recipients initiated in earlier years; 15% of the e-GAHT sample initiated between 2013 and 2015, compared to just 4% of the t-GAHT sample. Compared to people accessing e-GAHT, t-GAHT recipients used more mental health services (3.08 vs 1.59 per annum) and mental health prescriptions (2.28 vs 1.59 prescriptions) before initiating GAHT.

**Table 1:** Descriptive statistics.

	Testosterone-based GAHT (11,883)	Oestradiol-based GAHT (20,358)
	Mean (SD)/%	Mean (SD)/%
Age first start GAHT	24.98 (21.75)	33.82 (17.85)
*Age group start GAHT*		
15–24 years	66.60%	45.55%
≥25 years	33.40%	54.45%
*Year start GAHT*		
2013	0.66%	5.10%
2014	0.70%	4.74%
2015	2.08%	4.87%
2016	3.27%	5.67%
2017	5.23%	5.41%
2018	8.16%	6.18%
2019	9.47%	7.26%
2020	9.44%	8.30%
2021	11.93%	10.34%
2022	14.37%	12.88%
2023	17.06%	14.30%
2024	17.63%	14.93%
Years observed post GAHT	4.09 (2.59)	4.87 (3.28)
*Mental healthcare use*		
High baseline mental healthcare engagement^a^	41.01%	30.43%
Number of mental health services per annum (all years pre GAHT)	3.08 (3.90)	1.59 (2.66)
Number of mental health prescriptions per annum (all years pre GAHT)	2.58 (3.94)	1.59 (3.65)
Number of mental health services per annum^b^ (three years pre GAHT)	3.56 (6.26)	3.00 (4.12)
Number of mental health prescriptions per annum^b^ (three years pre GAHT)	1.72 (5.54)	1.74 (4.66)

Notes: GAHT = Gender affirming hormone therapy.

a High baseline engagement based on whether individuals' annual use of mental health prescriptions was above the average for their regimen in the years before the reference period.

b Three years pre GAHT corresponds to the reference period.

### Event study results

**Fig. 1:**
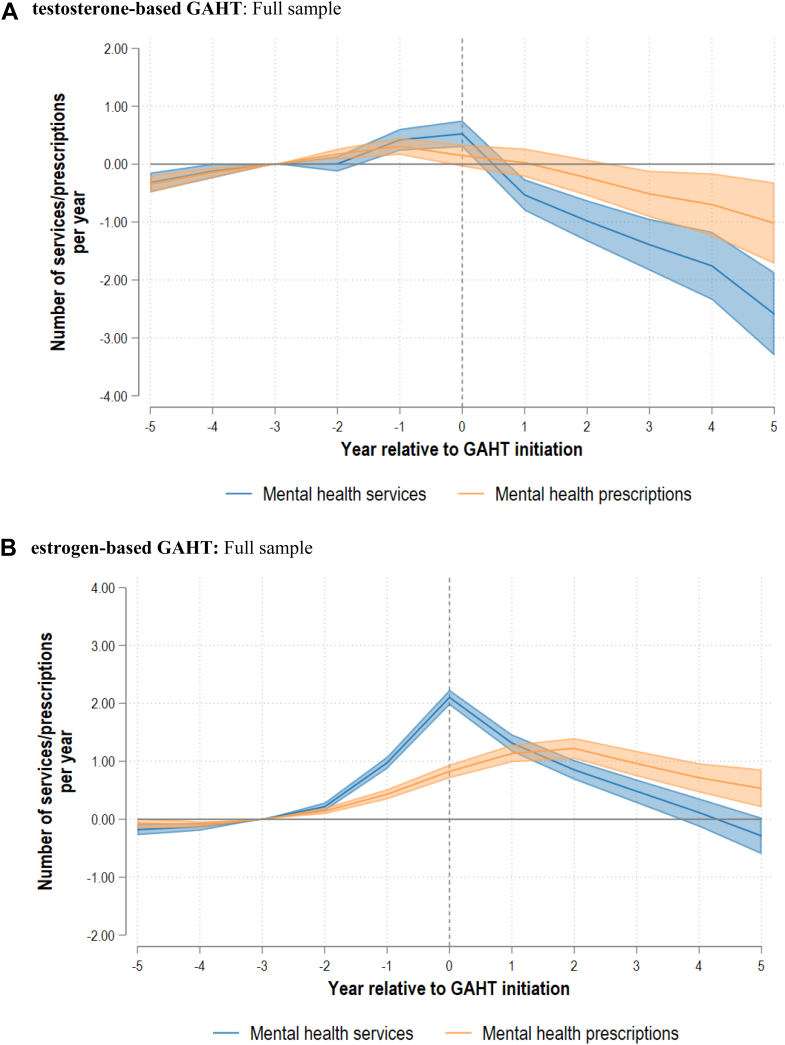
*Mental healthcare utilisation relative to GAHT initiation by regimen type*. **A) testosterone-based GAHT**: Full sample. **B) estrogen-based GAHT:** Full sample. **Notes:** GAHT = Gender affirming hormone therapy. All coefficients are referenced to event time three years prior to initiation.

**Fig. 2:**
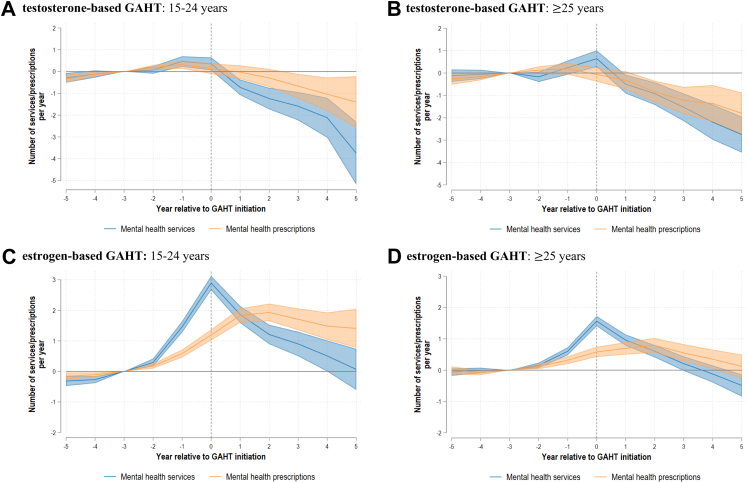
*Mental healthcare utilisation relative to GAHT initiation by regimen type and age group*. **A) testosterone-based GAHT**: 15–24 years. **B) testosterone-based GAHT**: ≥ 25 years. **C) estrogen-based GAHT**: 15–24 years. **D) estrogen-based GAHT**: ≥ 25 years. Notes: GAHT = Gender affirming hormone therap. All coefficients are referenced to event time three years prior to initiation.

**Fig. 3:**
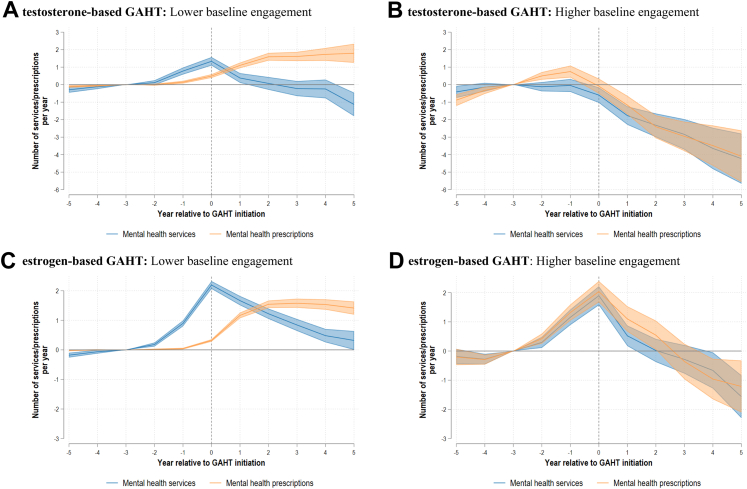
*Mental healthcare utilisation relative to GAHT initiation by regimen type and baseline mental healthcare engagement*. **A) testosterone-based GAHT**: Lower baseline engagement. **B) testosterone-based GAHT**: Higher baseline engagement. **C) estrogen-based GAHT**: Lower baseline engagement. **D) estrogen-based GAHT**: Higher baseline engagement. Notes: GAHT = Gender affirming hormone therapy. All coefficients are referenced to event time three years prior to initiation. Lower (higher) baseline engagement defined as whether below (above) group mean utilization of mental health prescriptions in all periods prior to the reference period (i.e., all event time ≤ −3).

The main event-study results are presented in Fig. 1. These figures show the average within-individual change in use of mental health services and mental health prescriptions for each year by regimen type relative to the baseline period (three years before GAHT initiation). Values represent absolute (arithmetic) changes; that is, the average number of additional or fewer services or prescriptions per person per year relative to baseline. Figs. 2 and 3 respectively show the results stratified by age and baseline mental healthcare engagement. Full model outputs are provided in Supplementary Materials SM3–SM6.

For t-GAHT recipients, mental healthcare use initially increased in the year of initiation by +0.52 (95% CI 0.29; 0.75) more services per person relative to baseline. The corresponding estimate for prescriptions was +0.15 (95% CI −0.04; 0.34). While the point estimate suggests a modest increase, this change is not statistically distinguishable from baseline use in that year.

This was succeeded by a sustained reduction in mental healthcare use and at five years after initiation, relative to baseline, t-GAHT recipients used fewer mental health services [−2.59 (95% CI −3.31; −1.87)] and mental health prescriptions [−1.02 (95% CI −1.72; −0.31)], corresponding to 73% and 34% reductions respectively. Reductions in mental health services were slightly more pronounced among younger t-GAHT recipients, albeit the overlap in confidence intervals suggests these differences were not statistically significant (Fig. 2).

Among e-GAHT recipients, mental health service use steadily increased up to the year of initiation [+2.10 (95% CI 1.97; 2.24)], then declined sharply. Five years after initiation, mental health service use among e-GAHT recipients was not significantly different relative the reference period [−0.29 (95% CI −0.60; 0.03)]. Following e-GAHT initiation, use of mental health prescriptions increased for up to two years [+1.22 (95% CI 1.04; 1.40) after two years]—and declined thereafter. Compared to baseline, e-GAHT recipients used slightly more scripts after five years [+0.53 (95% CI 0.20; 0.86)].

The uptick in mental health service use in the year of initiation was larger for younger e-GAHT recipients [+2.89 (95% CI 2.66; 3.13) among 15–24 year olds] compared to those initiating at 25 years and above [+1.56 (95% CI 1.40; 1.73)]. Similarly, the increase in mental health prescriptions was more prominent for younger e-GAHT recipients. For individuals initiating e-GAHT at 25 years and above, use of prescription medication for mental health conditions was not significantly different to the reference period after five years [+0.12 (95% CI −0.26; 0.50)].

Individuals with higher levels of baseline mental healthcare engagement saw the largest reductions in mental health services and prescriptions, with similar patterns observed for both e-GAHT and t-GAHT recipients (Fig. 3). After five years, t-GAHT and e-GAHT recipients with higher baseline engagement used fewer mental health services [−4.23 (95% CI −5.66; −2.80) and −1.57 (95% CI −2.30; −0.83), respectively] and fewer mental health prescriptions [−4.09 (95% CI −5.57; −2.62) and −1.22 (95% CI −2.11; −0.33), respectively]. For individuals with lower initial baseline engagement, reductions in mental health service use were less pronounced [−1.13 (95% CI −1.80; −0.46) for t-GAHT and +0.32 (95% CI 0.00; 0.64) for e-GAHT at five years relative to baseline]. Use of mental health prescriptions also increased for people with lower engagement relative to baseline [+1.79 (95% CI 1.25; 2.33) for t-GAHT and 1.41 (95% CI 1.19; 1.64) for e-GAHT at five years].

## Discussion

In this paper, we estimated the utilisation of mental health services and mental health prescriptions following initiation of GAHT among 32,241 trans Australians. We find that for both t-GAHT and e-GAHT recipients, mental healthcare use initially increases around initiation but then reduces significantly, with larger reductions for t-GAHT recipients and individuals with higher baseline mental healthcare engagement. Mental healthcare use patterns were broadly similar for younger (15–24 years) and older (≥25 years) t-GAHT recipients. However, reductions in mental health prescriptions were more pronounced among e-GAHT recipients who initiated treatment at 25 years or older, compared to those who initiated at 15–24 years.

The increased mental health service use in the periods immediately prior to, and coinciding with, GAHT initiation, is consistent with treatment guidelines recommending concurrent mental health support during access to GAHT.^6^ The observed reductions in mental health service use following GAHT initiation significantly add to the evidence suggesting that GAHT improves mental health and wellbeing among trans people accessing this care.^8^^,^^11^

The heterogeneous effects in the use of mental health prescriptions by regimen could be explained by several factors. First, one may expect that medically transitioning could lead to different experiences for t-GAHT compared to e-GAHT recipients. Several studies have found that oestradiol is associated with smaller changes in gender dysphoria and mental health outcomes, particularly in the short-term.^9^ Transitioning has also been linked to greater societal stigma and discrimination for transfeminine individuals relative to transmasculine individuals, including more substantial earnings penalties.^22^^,^^24^

The less pronounced reductions in mental health treatment among younger GAHT initiators may reflect both the additional monitoring recommended for this age group,^6^ but also broader age-related patterns in Australia. Use of mental health services and prescriptions is markedly higher among Australians aged 15–24 years compared with older adults.^25^ This suggests that some of the elevated engagement we observe may reflect underlying age-related patterns in help-seeking rather than GAHT-specific dynamics.

Our results do however suggest that unmet need for mental health treatment plays an important role for mental healthcare demand around GAHT initiation. For instance, we observe that increased utilisation of mental health prescriptions among GAHT recipients was driven by those with lower initial levels of mental healthcare engagement. Several studies have shown that transfeminine individuals experience poorer mental health outcomes—particularly, higher levels of suicidal ideation and suicidality—compared to transmasculine individuals.^18^^,^^26^ Given e-GAHT recipients had lower use of mental healthcare than t-GAHT recipients prior to GAHT initiation, the uptick in mental health prescriptions among individuals initiating e-GAHT could therefore reflect the emergence or identification of previously unmet mental health needs, consistent with treatment engagement rather than deterioration of mental health.

These patterns are also consistent with literature emphasising the role of gendered social norms and the social construction of mental health in shaping help-seeking, particularly among people assigned male at birth. Previous studies suggest that individuals assigned male at birth are socialised to minimise distress, avoid formal help-seeking, and perceive mental healthcare as more stigmatising, relative to individuals assigned female at birth.^27^^,^^28^ Such early-life socialisation may continue to influence patterns of mental healthcare engagement regardless of later gender identity, potentially contributing to the lower baseline mental healthcare utilisation observed among e-GAHT recipients.

Our results differ from the two earlier, smaller studies in Sweden and the US which did not find a significant association with GAHT initiation and mental healthcare visits.^12^^,^^13^ However, our finding of increased mental health prescriptions initially following e-GAHT initiation does align with earlier US and Danish studies, which reported an association between hormone therapy and increased odds of psychotropic medication use.^13^^,^^14^ It is important to note that several factors, including time varying confounders, differences in institutional settings, and empirical designs complicate such comparisons. In particular, our study suggests that dynamics relative to initiation as well as heterogeneous effects by regimen, baseline engagement, and age at initiation are all important factors to consider. That is, the magnitude and direction of post-initiation changes differ meaningfully across subgroups—t-GAHT vs e-GAHT recipients, those with higher vs lower baseline mental healthcare use, and younger vs older initiators.

Our results should be considered with the context of several limitations. First, it is important to note that mental healthcare utilisation does not directly capture underlying need for mental healthcare. Individuals may require care but be unable or unwilling to access it, and reductions in mental healthcare use could, in some cases, reflect disengagement rather than improvements in mental health. However, given GAHT is linked to substantial improvements in self-reported mental health,^7^, ^8^, ^9^ this interpretation may be less likely.

Further, a key limitation of the administrative data used in this study is that we can only identify trans people who initiated GAHT but not individuals who desired to, but could not access, GAHT. It is also likely that there is selection among trans people that received GAHT, including differences in earlier vs later access. For example, those under 18 years of age who do not have family support will not be able to receive GAHT. The scarce supply of gender affirming clinics and clinicians providing GAHT in Australia is also likely to impact access, particularly for people living in rural and remote areas.^29^

In addition, the sample likely includes a small number of individuals with differences in sexual development and cisgender women prescribed testosterone for hypoactive sexual desire disorder; however, because this indication is usually treated with unsubsidised low-dose formulations in Australia, their representation in this data is expected to be minimal.^17^

As the Australian Census and the medical records within PLIDA do not currently capture information on gender identity, it is difficult to compare our results to the broader trans population. Nevertheless, the proportion of the population in this database that was identified as trans in this data aligns with other population samples of trans people accessing GAHT.^30^, ^31^, ^32^ Efforts to embed questions on gender identity in population level datasets, such as the Census, would contribute to a better understanding around the potential barriers to accessing GAHT.

It is also important to note that GAHT is only one aspect of gender affirming medical care and, due to data limitations, we cannot disentangle the mental health treatment effects of GAHT initiation from other types of care, such as puberty blockers. Similarly, many factors contributing to psychological vulnerability may not be directly influenced by GAHT. Future work should consider other important outcomes such as daily functioning, social and economic participation, and broader resource utilisation patterns, including hospitalisations.

While registry-based quasi-experimental designs such as the one used in this study have inherent limitations, they also provide rare opportunities to evaluate real-world outcomes at scale, where randomised trials would be neither feasible nor ethical. As such data become increasingly available, these approaches will continue to play an important role in strengthening the evidence base for gender-affirming care, particularly when combined with complementary outcome measures. For example, similar empirical analyses using administrative data have been used to examine total health system costs associated with GAHT initiation^33^ and gender-affirming surgeries.^34^ Together, such studies demonstrate the value of leveraging routinely collected administrative data to inform evidence-based policy and clinical practice.

Despite these limitations, our study has several strengths and unique contributions. It is, to our knowledge, the largest population-based study of GAHT recipients globally and the first to provide population-level evidence on the dynamics of mental healthcare relative to GAHT initiation, with robust estimates by regimen and for different age groups. Our empirical framework addresses biases present in studies that compare trans populations to cisgender samples by accounting for underlying differences in mental healthcare needs, including diverging baseline levels and trends in mental health treatment use.^35^ Using administrative data also allows us to address potential recall bias associated with using self-reported measures of healthcare use, including robust details on the timing of GAHT initiation.^10^

These results have important implications for social and health policy. In the context of recent attempts to restrict access to gender-affirming care, both internationally and within Australia,^36^^,^^37^ this study adds to a growing body of evidence highlighting the positive mental health impacts of GAHT.

## Contributors

KS, TB, BJN, and CSC conceptualised the study and methodology. KS and BJN acquired funding. KS conducted formal analysis and project administration. BJN accessed and verified the data. KS wrote the first draft of the manuscript. Interpretations of the results were made by KS, BJN, CC, TB, and CSC. All authors reviewed the analyses and drafts of this manuscript and approved its final version.

## Data sharing statement

Data is available upon request to the Australian Bureau of Statistics.

## Declaration of interests

KS is a cofounder, and serves on the committee, of LGBTQ + Economists and Allies in the Asia–Pacific (LEAP).

## References

[1] Erlangsen A., Jacobsen A.L., Ranning A., Delamare A.L., Nordentoft M., Frisch M. (2023). Transgender identity and suicide attempts and mortality in Denmark. JAMA.

[2] Watkinson R., Linfield A., Tielemans J., Francetic I., Munford L. (2023). Gender-related self-reported mental health inequalities in primary care in England: cross-sectional analysis using the GP patient survey. Lancet Public Health.

[3] Bränström R., Stormbom I., Bergendal M., Pachankis J.E. (2022). Transgender-based disparities in suicidality: a population-based study of key predictions from four theoretical models. Suicide Life Threat Behav.

[4] Dhejne C., Van Vlerken R., Heylens G., Arcelus J. (2016). Mental health and gender dysphoria: a review of the literature. Int Rev Psychiatry.

[5] Kim H.-H., Thayer N., Bernstein C., Cruz R., Roby C., Keuroghlian A.S. (2025). On the frontlines: protecting and advancing gender-affirming care in a hostile sociopolitical environment. J Gen Intern Med.

[6] Cheung A.S., Wynne K., Erasmus J., Murray S., Zajac J.D. (2019). Position statement on the hormonal management of adult transgender and gender diverse individuals. Med J Aust.

[7] Turban J.L., King D., Kobe J., Reisner S.L., Keuroghlian A.S. (2022). Access to gender-affirming hormones during adolescence and mental health outcomes among transgender adults. PLoS One.

[8] Nolan B.J., Zwickl S., Locke P., Zajac J.D., Cheung A.S. (2023). Early access to testosterone therapy in transgender and gender-diverse adults seeking masculinization: a randomized clinical trial. JAMA Netw Open.

[9] Foster Skewis L., Bretherton I., Leemaqz S.Y., Zajac J.D., Cheung A.S. (2021). Short-term effects of gender-affirming hormone therapy on dysphoria and quality of life in transgender individuals: a prospective controlled study. Front Endocrinol.

[10] Campbell T., Mann S., Nguyen D.H., Rodgers Y.V.D.M. (2023). Hormone therapy, suicidal risk, and transgender youth in the United States. AEA Pap Proc.

[11] Mann S., Campbell T., Nguyen D.H. (2024). Access to gender-affirming care and transgender mental health: evidence from medicaid coverage. Am J Health Econ.

[12] Bränström R., Pachankis J.E. (2020). Reduction in mental health treatment utilization among transgender individuals after gender-affirming surgeries: a total population study. Am J Psychiatry.

[13] Hisle-Gorman E., Schvey N.A., Adirim T.A. (2021). Mental healthcare utilization of transgender youth before and after affirming treatment. J Sex Med.

[14] Glintborg D., Møller J.-J.K., Rubin K.H. (2023). Gender-affirming treatment and mental health diagnoses in Danish transgender persons: a nationwide register-based cohort study. Eur J Endocrinol.

[15] ABS (2022). Person Level Integrated Data Asset (PLIDA). https://www.abs.gov.au/about/data-services/data-integration/integrated-data/person-level-integrated-data-asset-plida.

[16] Nolan B.J., Zwickl S., Zajac J.D., Cheung A.S. (2024). Gender affirmation testosterone therapy, Australia, 2021–22: a review of PBS dispensing data. Med J Aust.

[17] Saxby K., Nolan B.J. (2025). Temporal trends in gender-affirming hormone therapy initiation: evidence from whole-of-population Australian administrative data. Intern Med J.

[18] de Blok C.J., Wiepjes C.M., van Velzen D.M. (2021). Mortality trends over five decades in adult transgender people receiving hormone treatment: a report from the Amsterdam cohort of gender dysphoria. Lancet Diabetes Endocrinol.

[19] Malhi G.S., Bassett D., Boyce P. (2015). Royal Australian and New Zealand College of Psychiatrists clinical practice guidelines for mood disorders. Aust N Z J Psychiatry.

[20] Saxby K., Buchmueller T., de New S.C., Petrie D. (2025). Regional variation in mental healthcare utilization and suicide: evidence from movers in Australia. J Health Econ.

[21] De Chaisemartin C., D'Haultfœuille X. (2020). Two-way fixed effects estimators with heterogeneous treatment effects. Am Econ Rev.

[22] Carpenter C.S., Goodman L., Lee M.J. (2024).

[23] World Health Organization (2025). Adolescent health. https://www.who.int/southeastasia/health-topics/adolescent-health.

[24] Campbell T, Compton O, Dogan F, Kara Y, Nettuno L, Saxby K (2025). “Transgender Economics.” Available at SSRN 5491507. https://papers.ssrn.com/sol3/papers.cfm?abstract_id=5491507.

[25] AIHW (2024). Australian Institute of Health and Welfare.

[26] Wiepjes C.M., den Heijer M., Bremmer M.A. (2020). Trends in suicide death risk in transgender people: results from the Amsterdam Cohort of Gender Dysphoria study (1972–2017). Acta Psychiatr Scand.

[27] Addis M.E., Mahalik J.R. (2003). Men, masculinity, and the contexts of help seeking. Am Psychol.

[28] Seidler Z.E., Dawes A.J., Rice S.M., Oliffe J.L., Dhillon H.M. (2016). The role of masculinity in men's help-seeking for depression: a systematic review. Clin Psychol Rev.

[29] Saxby K., Stephens M. (2024). Medicare and priority populations: structural and place-based considerations for aboriginal and torres strait islander peoples and LGBTIQ+ Australians. Aust Econ Rev.

[30] Coleman E., Radix A.E., Bouman W.P. (2022). Standards of care for the health of transgender and gender diverse people, version 8. Int J Transgend Health.

[31] Baker K., Restar A. (2022). Utilization and costs of gender-affirming care in a commercially insured transgender population. J Law Med Ethics.

[32] Wiepjes C.M., Nota N.M., de Blok C.J. (2018). The Amsterdam cohort of gender dysphoria study (1972–2015): trends in prevalence, treatment, and regrets. J Sex Med.

[33] Saxby K., Petrie D., Nolan B.J. (2025).

[34] Saxby K., Nolan B.J. (2026). Mental health treatment following gender-affirming surgeries: evidence from administrative data in Australia. Int J of Transgend Health.

[35] Saxby K., Hutchinson-Tovar S., Bishop G.M. (2025). Gender identity and mental health inequalities 2001-2022: population-level evidence from an Australian cohort study. BMJ Ment Health.

[36] Alibudbud R. (2025). LGBTQ+ rights and health: a shifting landscape. Lancet.

[37] Queensland Government (2025). Media statement: Independent review into puberty blockers. https://statements.qld.gov.au/statements/101903.

