# Supplementary material for: Mental health treatment among transgender and gender diverse people following gender affirming hormone therapy: evidence from whole-of-population Australian administrative data
Source: eClinicalMedicine. 2026 Feb 2;92:103765. doi: 10.1016/j.eclinm.2026.103765 (PMC12947644; doi:10.1016/j.eclinm.2026.103765)
Supplement: Supplementary Material [file mmc1.docx]

# Supplementary Material

### Event study difference-in-difference model

We follow the approach by De Chaisemartin and d'Haultfoeuille ^1^ to estimate dynamic treatment effects with staggered treatment adoption. Our baseline specification is as follows:

| $Y_{it}=\alpha+\sum_{k=-5, k\neq-3}^{5} \beta_{l}D_{k,it}+\mu_{i}+\tau_{y}+\in_{it}$ | (1) |
| --- | --- |

where $Y$ denotes mental healthcare use (number of mental health services, mental health prescriptions) for individual $i$ within period $t$, $D_{k}$ are event time indicators (leads and lags) for time relative to GAHT initiation ($k=t-G_{i}$ , where $G_{i}$ is the GAHT initiation period for individual $i$), $\mu_{i}$ are individual fixed effects (which accounts for all observed and unobserved time-invariant confounders), $\tau_{y}$ are calendar year fixed effects, and $\in_{it}$ is the error term. The model is estimated separately for t-GAHT and e-GAHT recipients and weighted to account for the distribution of treatment initiation across individuals. This approach avoids negative treatment weights.^1^ Standard errors are clustered at the individual level, the level at which the treatment occurs.

### Event study results by regimen, full sample

#### Table A. 1 – Mental health services

| **Event time** | **Testosterone-based GAHT** | | | **Oestradiol-based GAHT** | | |
| --- | --- | --- | --- | --- | --- | --- |
|  | **beta** | **lower limit** | **upper limit** | **beta** | **lower limit** | **upper limit** |
| -5 | -0.32 | -0.49 | -0.15 | -0.18 | -0.28 | -0.08 |
| -4 | -0.12 | -0.24 | 0.01 | -0.13 | -0.20 | -0.06 |
| -3 | ref | ref | ref | ref | ref | ref |
| -2 | 0.01 | -0.13 | 0.14 | 0.22 | 0.14 | 0.29 |
| -1 | 0.42 | 0.23 | 0.61 | 0.98 | 0.87 | 1.08 |
| 0 | 0.52 | 0.29 | 0.75 | 2.10 | 1.97 | 2.24 |
| 1 | -0.53 | -0.81 | -0.26 | 1.32 | 1.16 | 1.47 |
| 2 | -0.98 | -1.34 | -0.62 | 0.85 | 0.68 | 1.02 |
| 3 | -1.39 | -1.84 | -0.94 | 0.48 | 0.28 | 0.69 |
| 4 | -1.76 | -2.35 | -1.17 | 0.12 | -0.13 | 0.36 |
| 5 | -2.59 | -3.31 | -1.87 | -0.29 | -0.60 | 0.03 |

Notes: All percentage changes are calculated relative to the reference mean at three years prior to GAHT initiation (i.e., event time=-3). The lower and upper limits represent the bounds of the 95% confidence interval.

#### Table A. 2 – Mental health prescriptions

| **Event time** | **Testosterone-based GAHT** | | | **Oestradiol-based GAHT** | | |
| --- | --- | --- | --- | --- | --- | --- |
|  | **beta** | **lower limit** | **upper limit** | **beta** | **lower limit** | **upper limit** |
| -5 | -0.35 | -0.49 | -0.20 | -0.07 | -0.16 | 0.02 |
| -4 | -0.14 | -0.23 | -0.05 | -0.09 | -0.14 | -0.04 |
| -3 | ref | ref | ref | ref | ref | ref |
| -2 | 0.17 | 0.08 | 0.27 | 0.14 | 0.09 | 0.20 |
| -1 | 0.31 | 0.16 | 0.46 | 0.43 | 0.34 | 0.52 |
| 0 | 0.15 | -0.04 | 0.34 | 0.83 | 0.71 | 0.95 |
| 1 | 0.03 | -0.22 | 0.27 | 1.14 | 0.98 | 1.29 |
| 2 | -0.23 | -0.55 | 0.08 | 1.22 | 1.04 | 1.40 |
| 3 | -0.51 | -0.91 | -0.11 | 0.96 | 0.74 | 1.18 |
| 4 | -0.70 | -1.24 | -0.16 | 0.72 | 0.46 | 0.97 |
| 5 | -1.02 | -1.72 | -0.31 | 0.53 | 0.20 | 0.86 |

Notes: All percentage changes are calculated relative to the reference mean at three years prior to GAHT initiation (i.e., event time=-3). The lower and upper limits represent the bounds of the 95% confidence interval.

### Event study results by regimen, 15-24 years at initiation

#### Table A. 3 – Mental health services

| **Event time** | **Testosterone-based GAHT** | | | **Oestradiol-based GAHT** | | |
| --- | --- | --- | --- | --- | --- | --- |
|  | **beta** | **lower limit** | **upper limit** | **beta** | **lower limit** | **upper limit** |
| -5 | -0.29 | -0.51 | -0.07 | -0.31 | -0.47 | -0.15 |
| -4 | -0.11 | -0.27 | 0.05 | -0.27 | -0.38 | -0.15 |
| -3 | ref | ref | ref | ref | ref | ref |
| -2 | 0.07 | -0.09 | 0.24 | 0.29 | 0.17 | 0.42 |
| -1 | 0.46 | 0.22 | 0.70 | 1.46 | 1.28 | 1.64 |
| 0 | 0.35 | 0.06 | 0.65 | 2.89 | 2.66 | 3.13 |
| 1 | -0.72 | -1.08 | -0.37 | 1.86 | 1.59 | 2.14 |
| 2 | -1.24 | -1.73 | -0.75 | 1.21 | 0.89 | 1.53 |
| 3 | -1.59 | -2.24 | -0.94 | 0.90 | 0.50 | 1.30 |
| 4 | -2.12 | -3.03 | -1.21 | 0.51 | 0.00 | 1.01 |
| 5 | -3.75 | -5.19 | -2.31 | 0.07 | -0.61 | 0.74 |

Notes: All percentage changes are calculated relative to the reference mean at three years prior to GAHT initiation (i.e., event time=-3). The lower and upper limits represent the bounds of the 95% confidence interval.

#### Table A. 4 – Mental health prescriptions

| **Event time** | **Testosterone-based GAHT** | | | **Oestradiol-based GAHT** | | |
| --- | --- | --- | --- | --- | --- | --- |
|  | **beta** | **lower limit** | **upper limit** | **beta** | **lower limit** | **upper limit** |
| -5 | -0.33 | -0.51 | -0.15 | -0.16 | -0.28 | -0.04 |
| -4 | -0.12 | -0.22 | -0.01 | -0.11 | -0.18 | -0.04 |
| -3 | ref | ref | ref | ref | ref | ref |
| -2 | 0.18 | 0.07 | 0.30 | 0.18 | 0.10 | 0.26 |
| -1 | 0.32 | 0.14 | 0.51 | 0.59 | 0.46 | 0.71 |
| 0 | 0.13 | -0.11 | 0.37 | 1.19 | 1.02 | 1.37 |
| 1 | -0.03 | -0.34 | 0.28 | 1.83 | 1.60 | 2.07 |
| 2 | -0.29 | -0.70 | 0.12 | 1.93 | 1.65 | 2.22 |
| 3 | -0.67 | -1.22 | -0.11 | 1.71 | 1.35 | 2.06 |
| 4 | -1.04 | -1.80 | -0.28 | 1.48 | 1.04 | 1.93 |
| 5 | -1.40 | -2.59 | -0.21 | 1.41 | 0.77 | 2.04 |

Notes: All percentage changes are calculated relative to the reference mean at three years prior to GAHT initiation (i.e., event time=-3). The lower and upper limits represent the bounds of the 95% confidence interval.

### Event study results by regimen, $\boldsymbol{\geq}$25 years at initiation

#### Table A. 5 – Mental health services

| **Event time** | **Testosterone-based GAHT** | | | **Oestradiol-based GAHT** | | |
| --- | --- | --- | --- | --- | --- | --- |
|  | **beta** | **lower limit** | **upper limit** | **beta** | **lower limit** | **upper limit** |
| -5 | -0.12 | -0.39 | 0.16 | -0.06 | -0.18 | 0.06 |
| -4 | -0.06 | -0.26 | 0.14 | -0.01 | -0.10 | 0.08 |
| -3 | ref | ref | ref | ref | ref | ref |
| -2 | -0.17 | -0.40 | 0.05 | 0.15 | 0.07 | 0.24 |
| -1 | 0.24 | -0.07 | 0.55 | 0.60 | 0.48 | 0.73 |
| 0 | 0.64 | 0.27 | 1.01 | 1.56 | 1.40 | 1.73 |
| 1 | -0.50 | -0.91 | -0.08 | 0.96 | 0.78 | 1.14 |
| 2 | -0.92 | -1.42 | -0.42 | 0.60 | 0.41 | 0.80 |
| 3 | -1.54 | -2.15 | -0.92 | 0.21 | -0.02 | 0.44 |
| 4 | -2.20 | -2.98 | -1.41 | -0.12 | -0.39 | 0.15 |
| 5 | -2.75 | -3.55 | -1.95 | -0.49 | -0.84 | -0.14 |

Notes: GAHT=Gender affirming hormone therapy. The lower and upper limits represent the bounds of the 95% confidence interval.

#### Table A. 6 – Mental health prescriptions

| **Event time** | **Testosterone-based GAHT** | | | **Oestradiol-based GAHT** | | |
| --- | --- | --- | --- | --- | --- | --- |
|  | **beta** | **lower limit** | **upper limit** | **beta** | **lower limit** | **upper limit** |
| -5 | -0.27 | -0.52 | -0.01 | -0.01 | -0.13 | 0.12 |
| -4 | -0.16 | -0.32 | 0.00 | -0.08 | -0.16 | 0.00 |
| -3 | ref | ref | ref | ref | ref | ref |
| -2 | 0.13 | -0.04 | 0.29 | 0.12 | 0.04 | 0.19 |
| -1 | 0.18 | -0.07 | 0.44 | 0.32 | 0.20 | 0.44 |
| 0 | -0.07 | -0.38 | 0.25 | 0.58 | 0.42 | 0.74 |
| 1 | -0.32 | -0.72 | 0.07 | 0.70 | 0.50 | 0.89 |
| 2 | -0.84 | -1.32 | -0.35 | 0.79 | 0.56 | 1.02 |
| 3 | -1.22 | -1.82 | -0.63 | 0.55 | 0.27 | 0.83 |
| 4 | -1.36 | -2.18 | -0.54 | 0.35 | 0.05 | 0.65 |
| 5 | -1.80 | -2.73 | -0.87 | 0.12 | -0.26 | 0.50 |

Notes: GAHT=Gender affirming hormone therapy. The lower and upper limits represent the bounds of the 95% confidence interval.

### Event study results for individuals with lower baseline mental healthcare engagement

#### Table A. 7 – Mental health services

| **Event time** | **Testosterone-based GAHT** | | | **Oestradiol-based GAHT** | | |
| --- | --- | --- | --- | --- | --- | --- |
|  | **beta** | **lower limit** | **upper limit** | **beta** | **lower limit** | **upper limit** |
| -5 | -0.29 | -0.46 | -0.13 | -0.18 | -0.26 | -0.10 |
| -4 | -0.12 | -0.25 | 0.01 | -0.06 | -0.12 | 0.00 |
| -3 | ref | ref | ref | ref | ref | ref |
| -2 | 0.11 | -0.03 | 0.25 | 0.18 | 0.11 | 0.25 |
| -1 | 0.78 | 0.58 | 0.97 | 0.90 | 0.80 | 1.00 |
| 0 | 1.33 | 1.09 | 1.57 | 2.20 | 2.06 | 2.33 |
| 1 | 0.38 | 0.10 | 0.66 | 1.67 | 1.51 | 1.82 |
| 2 | 0.07 | -0.29 | 0.43 | 1.22 | 1.05 | 1.39 |
| 3 | -0.22 | -0.65 | 0.20 | 0.84 | 0.64 | 1.04 |
| 4 | -0.25 | -0.78 | 0.29 | 0.48 | 0.26 | 0.71 |
| 5 | -1.13 | -1.80 | -0.46 | 0.32 | 0.00 | 0.64 |

Notes: GAHT=Gender affirming hormone therapy. The lower and upper limits represent the bounds of the 95% confidence interval.

#### Table A. 8 – Mental health prescriptions

| **Event time** | **Testosterone-based GAHT** | | | **Oestradiol-based GAHT** | | |
| --- | --- | --- | --- | --- | --- | --- |
|  | **beta** | **lower limit** | **upper limit** | **beta** | **lower limit** | **upper limit** |
| -5 | -0.11 | -0.17 | -0.04 | -0.02 | -0.04 | 0.01 |
| -4 | -0.06 | -0.11 | -0.01 | 0.00 | -0.02 | 0.02 |
| -3 | ref | ref | ref | ref | ref | ref |
| -2 | 0.00 | -0.05 | 0.05 | 0.02 | 0.00 | 0.04 |
| -1 | 0.14 | 0.07 | 0.21 | 0.04 | 0.02 | 0.07 |
| 0 | 0.49 | 0.39 | 0.59 | 0.32 | 0.28 | 0.36 |
| 1 | 1.10 | 0.94 | 1.26 | 1.17 | 1.07 | 1.26 |
| 2 | 1.59 | 1.38 | 1.81 | 1.54 | 1.42 | 1.67 |
| 3 | 1.61 | 1.35 | 1.87 | 1.58 | 1.43 | 1.73 |
| 4 | 1.73 | 1.37 | 2.09 | 1.54 | 1.36 | 1.71 |
| 5 | 1.79 | 1.25 | 2.33 | 1.41 | 1.19 | 1.64 |

Notes: GAHT=Gender affirming hormone therapy. The lower and upper limits represent the bounds of the 95% confidence interval.

### Event study results for individuals with higher baseline mental healthcare engagement

#### Table A. 9 – Mental health services

| **Event time** | **Testosterone-based GAHT** | | | **Oestradiol-based GAHT** | | |
| --- | --- | --- | --- | --- | --- | --- |
|  | **beta** | **lower limit** | **upper limit** | **beta** | **lower limit** | **upper limit** |
| -5 | -0.42 | -0.76 | -0.08 | -0.19 | -0.45 | 0.07 |
| -4 | -0.14 | -0.39 | 0.10 | -0.28 | -0.46 | -0.10 |
| -3 | ref | ref | ref | ref | ref | ref |
| -2 | -0.12 | -0.38 | 0.14 | 0.30 | 0.11 | 0.48 |
| -1 | -0.05 | -0.41 | 0.32 | 1.16 | 0.89 | 1.42 |
| 0 | -0.60 | -1.04 | -0.16 | 1.90 | 1.57 | 2.22 |
| 1 | -1.77 | -2.29 | -1.24 | 0.52 | 0.17 | 0.87 |
| 2 | -2.32 | -3.00 | -1.65 | 0.02 | -0.38 | 0.41 |
| 3 | -2.84 | -3.71 | -1.97 | -0.29 | -0.77 | 0.20 |
| 4 | -3.65 | -4.82 | -2.47 | -0.67 | -1.28 | -0.05 |
| 5 | -4.23 | -5.66 | -2.80 | -1.57 | -2.30 | -0.83 |

Notes: GAHT=Gender affirming hormone therapy. The lower and upper limits represent the bounds of the 95% confidence interval.

#### Table A. 10 – Mental health prescriptions

| **Event time** | **Testosterone-based GAHT** | | | **Oestradiol-based GAHT** | | |
| --- | --- | --- | --- | --- | --- | --- |
|  | **beta** | **lower limit** | **upper limit** | **beta** | **lower limit** | **upper limit** |
| -5 | -0.88 | -1.22 | -0.55 | -0.20 | -0.48 | 0.07 |
| -4 | -0.32 | -0.53 | -0.12 | -0.29 | -0.46 | -0.12 |
| -3 | ref | ref | ref | ref | ref | ref |
| -2 | 0.50 | 0.28 | 0.72 | 0.42 | 0.25 | 0.60 |
| -1 | 0.75 | 0.41 | 1.09 | 1.32 | 1.04 | 1.59 |
| 0 | -0.08 | -0.51 | 0.34 | 2.04 | 1.67 | 2.40 |
| 1 | -1.17 | -1.70 | -0.64 | 1.10 | 0.67 | 1.53 |
| 2 | -2.41 | -3.06 | -1.76 | 0.54 | 0.04 | 1.04 |
| 3 | -2.96 | -3.79 | -2.12 | -0.36 | -0.97 | 0.25 |
| 4 | -3.48 | -4.63 | -2.33 | -0.96 | -1.66 | -0.27 |
| 5 | -4.09 | -5.57 | -2.62 | -1.22 | -2.11 | -0.33 |

Notes: GAHT=Gender affirming hormone therapy. The lower and upper limits represent the bounds of the 95% confidence interval.

# Supplementary Material References

1. De Chaisemartin C, d'Haultfoeuille X. Two-way fixed effects estimators with heterogeneous treatment effects. *American Economic Review* 2020; **110**(9): 2964-96.
